# Important Genes in the Pathogenesis of 5q- Syndrome and Their Connection with Ribosomal Stress and the Innate Immune System Pathway

**DOI:** 10.1155/2012/179402

**Published:** 2012-02-13

**Authors:** Ota Fuchs

**Affiliations:** ^1^ Institute of Hematology and Blood Transfusion, U Nemocnice 1, 128 20 Prague 2, Czech Republic; ^2^Center of Experimental Hematology, First Medical Faculty, Charles University, Institute of Pathological Physiology, 128 53 Prague 2, Czech Republic

## Abstract

Myelodysplastic syndrome (MDS) with interstitial deletion of a segment of the long arm of chromosome 5q [del(5q)] is characterized by bone marrow erythroid hyperplasia, atypical megakaryocytes, thrombocythemia, refractory anemia, and low risk of progression to acute myeloid leukemia (AML) compared with other types of MDS. The long arm of chromosome 5 contains two distinct commonly deleted regions (CDRs). The more distal CDR lies in 5q33.1 and contains 40 protein-coding genes and genes coding microRNAs (miR-143, miR-145). In 5q-syndrome one allele is deleted that accounts for haploinsufficiency of these genes. The mechanism of erythroid failure appears to involve the decreased expression of the ribosomal protein S14 (*RPS14*) gene and the upregulation of the p53 pathway by ribosomal stress. Friend leukemia virus integration 1 (Fli1) is one of the target genes of miR145. Increased Fli1 expression enables effective megakaryopoiesis in 5q-syndrome.

## 1. Introduction

 Approximately 15% of patients with MDS have abnormalities of chromosome 5 [1]. These abnormalities include interstitial deletion of a segment of the long arm of chromosome 5q [del(5q), 5q-syndrome], monosomy, and unbalanced translocations. 5q-syndrome as MDS category was defined by the World Health Organization (WHO) [2], and it is characterized by refractory macrocytic anemia with dyserythropoiesis, transfusion dependence, normal to elevated platelet counts, hypolobated and nonlobated megakaryocytes, female preponderance, a favourable prognosis, and low risk of progression to AML compared with other types of MDS [3–10]. Many research groups analysed chromosome 5q deletions in patients with 5q-syndrome. We will shortly describe these studies from historical point of view and not for relevance in the pathogenesis.

 Deletion of interferon regulatory factor-1 gene (*IRF1*) mapped to chromosome 5q31 was detected [11]. IRF1 is a putative tumor suppressor and a transcriptional activator of interferon and interferon-stimulated genes. *IRF1 *dosage experiments demonstrated that 2 patients with 5q-syndrome retained both copies of this gene [12]. Thus, *IRF1* maps outside the common deleted segment of the 5q-chromosome, and the same result was obtained in the case of *EGR1 *(epidermal growth receptor 1) [13, 14].

 Molecular mapping techniques defined the region of gene loss in two patients with the 5q-syndrome and uncharacteristically small 5q deletions (5q31–q33) [14]. The allelic loss of 10 genes localized to 5q23-qter [centromere-*CSF2 *(colony-stimulating factor 2/granulocyte-macrophage/)*-EGR1 *(early growth response1)*-FGFA *(fibroblast growth factor acidic)*-GRL *(glucocorticoid receptor)*-ADRB2 *(*β*-2 adrenergic receptor)*-CSF1R *(colony-stimulating factor 1)*-SPARC *(secreted protein, acidic, cysteine-rich/osteonectin/)*-GLUH1*(human glutamate receptor 1)*-NKSF1 *(NK-cell-stimulating factor chain 1)*-FLT4 *(Fms-related tyrosine kinase 4)-telomere] was investigated in peripheral blood cell fractions. Gene dosage experiments demonstrated that *CSF2*,* EGR1*,* NKSF1,* and *FLT4* were retained on the 5q-chromosome in both patients and that *FGFA* was retained in one patient, thus placing these genes outside the critical region. *GRL*,* ADRB2*,* CSF1R*,* SPARC*, and *GLUH1* were shown to be deleted in both patients. The proximal breakpoint is localized between *EGR1* and *FGFA* in one patient and between *FGFA* and *ADRB2* in the other, and the distal breakpoint is localized between *GLUH1* and *NKSF1* in both patients [14]. Pulsed-field gel electrophoresis was used to map the 5q deletion breakpoints, and breakpoint-specific fragments were detected with *FGFA* probe in the granulocyte but not the lymphocyte fraction of one patient. This study has established the critical region of gene loss of the 5q-chromosome in the 5q-syndrome, giving the location for a putative tumor-suppressor gene in the 5.6 Mb region between *FGFA* and *NKSF1* [14].

 Boultwood et al. [15] characterised the commonly deleted region (CDR) in a study involving sixteen 5q-syndrome patients to a 1.5 Mb interval located at 5q32-5q33 between D5S413 marker and *GLRA1* (glycine receptor subunit *α*-1). This region contains *PDE6A *(phosphodiesterase 6A), *CSF1R*, *CD74* (CD74/cluster of differentiation 74/molecule, major histocompatibility complex, class II invariant chain), *TCOF1 *(Treacher-Collins-Franceschetti syndrome 1), *ANXA6 *(annexin A6), *SPARC*, and *FAT2 *(FAT tummor suppressor homolog 2, also known as cadherin family member 8 or multiple epidermal growth factor-like domains protein 1) genes. Advanced MDS and AML had a large del(5q) which contained a distinct 5q31 CDR [16, 17].

 It was shown that MDS arises from the transformation of a multipotent hematopoietic stem cell (HSC) or myeloid-committed progenitor cell [18, 19]. These data suggest that the gene or genes that are inactivated in the 5q-syndrome will be expressed in normal hematopoietic stem and progenitor cells. The expression of all 40 genes assigned to the CDR by means of the Ensembl program was thus examined in normal human bone marrow CD34^+^ cells by means of RT-PCR. Of the 40 genes in the CDR, 33 were expressed in CD34^+^ cells and, therefore, represented candidate genes since they were expressed within the HSC/progenitor cell compartment [15].

**Figure 1:**
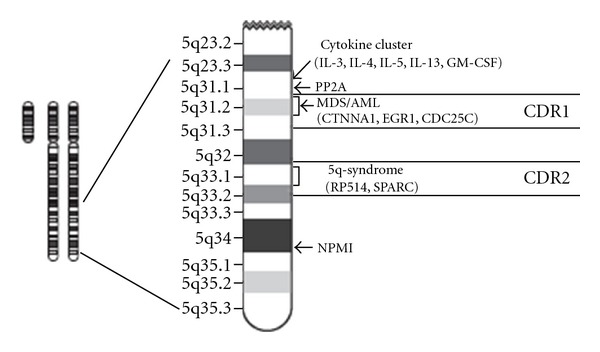
Schematic diagram of human chromosome 5q map showing commonly deleted regions.

 Cytogenetic mapping of commonly deleted regions (CDRs) centered on 5q31 and 5q32-5q33 identified candidate tumor-suppressor genes, including the transcription factor Egr1/Krox20 and the cytoskeletal remodeling protein, alpha-catenin on 5q31, and the ribosomal subunit protein RPS14 on 5q32-5q33 (Figure 1). Although each acts as a tumor suppressor, alone or in combination, no molecular mechanism accounts for how defects in individual 5q candidates may act as a lesion driving MDS or contributing to malignant progression in MPN (myeloproliferative neoplasms). One candidate gene that resides between the conventional del(5q)/5q-MDS-associated CDRs is DIAPH1 (5q31.3). DIAPH1 encodes the mammalian diaphanous-related formin, mDia1. mDia1 has critical roles in actin remodeling in cell division and in response to adhesive and migratory stimuli. Eisenmann et al. [20] examined evidence, with a focus on mouse gene-targeting experiments, that mDia1 acts as a node in a tumor-suppressor network that involves multiple 5q gene products.

## 2. The Possible Role of Candidate Genes from CDR in 5q-Syndrome

 All the 40 genes within the CDR were sequenced, and no mutations were found [21]. This finding suggests that 5q-syndrome may be a “one-hit” cancer in contrast to the “two-hit hypothesis” for the mechanism of cancer development described by Knudson [22]. Knudson's two-hit model of tumour suppressor genes supposes that two mutations are required to cause a tumour, one occurring in each of the two alleles of the gene. Many reports described candidate tumour suppressors that do not conform to this standard definition, including haploinsufficient genes requiring inactivation of only one allele and genes inactivated not by mutation but rather epigenetic hypermethylation [23]. Thus, a dosage effect as the result of the loss of a single allele of a gene (haploinsufficiency) may be responsible for the 5q-syndrome. Haploinsufficiency of multiple genes mapping to the CDR at 5q31-32 may contribute to the pathogenesis of 5q-syndrome [24–27]. Haploinsufficiency may be a driving force in cancer [28].

 Boultwood et al. [21] compared the transcriptome of the CD34^+^ cells in a group of 10 patients with the 5q-syndrome using the Affymetrix Gene Chip U133 Plus 2.0 array platform with the transcriptome of CD34^+^ cells from 16 healthy control subjects and 14 patients with refractory anemia and a normal karyotype. The majority of the genes assigned to the CDR of the 5q-syndrome at 5q31-q32 showed a reduction in expression levels in patients with the 5q-syndrome, consistent with the loss of one allele. Candidate genes showing haploinsufficiency in the 5q-syndrome included the tumour suppressor gene *SPARC* and *RPS14*, a component of the 40S ribosomal subunit. Two genes mapping to the CDR, *RBM22* (RNA-binding motif 22) and *CSNK1A1* (casein kinase 1, *α*1), showed more than 50% reduction in gene expression, consistent with the downregulation of the remaining allele. RBM22 plays a role in splicing and nuclear translocation of the calcium binding protein ALG2 (apoptosis-linked gene 2) and causes deregulation of apoptosis [29]. CSNK1A1 regulates the Hedgehog signal pathway which governs cell growth in cooperation with Wnt signaling [30, 31]. This study identified several significantly deregulated gene pathways in patients with the 5q-syndrome, and gene pathway analysis data supported the proposal that *SPARC* might play a role in the pathogenesis of the 5q-syndrome.

### 2.1. Role of SPARC in 5q-Syndrome

 Lehmann et al. [32] further studied the hematopoietic system in SPARC-null mice. These mice showed significantly lower platelet counts compared to wild-type animals. Although hemoglobin, hematocrit, and mean corpuscular volume (MCV) were lower in mice lacking SPARC, differences were not statistically significant. SPARC-null mice showed a significantly impaired ability to form erythroid burst-forming units (BFU-E). However, no significant differences were found in the formation of erythroid colony-forming units (CFU-E), granulocyte/monocyte colony-forming units (CFU-GM), or megakaryocyte colony-forming units (CFU-Mk) in these animals. These authors concluded that many of the genes within the CDR associated with the 5q-syndrome exhibit significantly decreased expression and that *SPARC*, as a potential tumor suppressor gene, may play some but not the key role in the pathogenesis of this disease.

### 2.2. RPS14 and Ribosomopathies

 Ebert et al. [33] used an RNA-mediated interference- (RNAi-) based approach to discovery of the 5q- disease gene. They found that partial loss of function of the ribosomal subunit protein RPS14 phenocopies the disease in normal haematopoietic progenitor cells. The forced expression of RPS14 with a lentiviral cDNA expression vector rescued the disease phenotype in patient-derived bone marrow cells. In addition, they identified a block in the processing of preribosomal RNA in RPS14-deficient cells. This block was functionally equivalent to the defect in Diamond-Blackfan anemia, linking the molecular pathophysiology of the 5q-syndrome to a congenital syndrome causing bone marrow failure [34]. These results indicate that the 5q-syndrome is caused by a defect in ribosomal protein function and suggest that RNAi screening is an effective strategy for identifying causal haploinsufficiency disease genes. Multiple different RPS14 shRNAs decreased the ratio of erythroid to megakaryocytic cells produced *in vitro*. These RPS14 shRNAs decreased also the ratio of erythroid to myeloid cells and caused the apoptosis of differentiating erythroid cells. The decreased level of *RPS14 *gene expression in 5q-syndrome is not caused by the aberrant methylation of the *RPS14 *gene promoter [35]. The 5q-syndrome belongs to ribosomopathies that are disorders in which genetic abnormalities cause impaired ribosome biogenesis and function [25].

 Inherited mutations for several ribosomal protein genes (*RPS19*,* RPS27A*,* RPS26*,* RPS24*,* RPS17*,* RPS15*,* RPS10*,* RPS7*,* RPL5*,* RPL11*,* RPL35a*, and *RPL36*) cause Diamond-Blackfan anemia (DBA, OMIM#205900) [34, 36–46]. Josephs [47] and Diamond and Blackfan [48] reported the first cases of this rare disease almost exclusively affecting the erythroid lineage. DBA is characterized by a moderate to severe anemia with normal neutrophil and platelet counts and a marked reduction in number of red cell precursors in an otherwise normocellular bone marrow. Several other bone marrow failure syndromes are also associated with defects in ribosome biogenesis and function [25, 38].

 A murine model of the human 5q-syndrome was developed in which haploinsufficiency of the *Cd74*-*Nid67* (CD74 antigen gene—nerve growth factor-induced differentiation clone 67 gene) region containing the *RPS14 *gene on mouse chromosome 18 maps CDR of the human 5q-syndrome and contains 8 known genes [49]. This was achieved using Cre-*loxP *recombination to delete this region. An *Lmo2-Cre* transgene was used to restrict the deletion to the hematopoietic compartment. Two genes (*Ndst1*/glucosaminyl N-deacetylase/N-sulfotransferase 1 gene/and *Cd74*) from this segment on mouse chromosome 18 have been excluded as candidate genes. *RPS14 *was the major candidate gene in relation to the phenotype [49]. Mice with one deleted allele displayed hypocellular bone marrow as a consequence of a defect in hematopoietic progenitor function that could be rescued by p53 inactivation. This murine model also displayed a macrocytic anemia with dysplastic features in the bone marrow and monolobulated megakaryocytes, common features of 5q-syndrome. These changes correlated with an increase in a population of highly p53 positive cells in the bone marrow and with elevated apoptosis (see Section 4).

**Figure 2:**
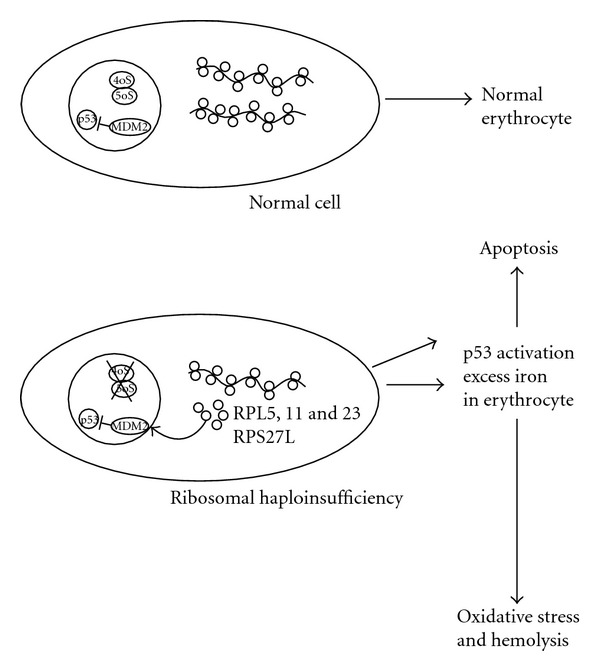
Ribosomal stress in 5q-syndrome. On the top is schematically shown normal erythroid cell with normal ribosome biogenesis and p53 levels. On the bottom is erythroid cell where p53 is activated by ribosomal stress secondary to RPS14 haploinsufficiency. Ribosomal proteins RPL5, 11, 23, and RPS27L bind HDM2 and activate p53. The ribosomal protein S27-like (RPS27L) is induced by p53-activating signals and promotes apoptosis [53, 54]. To date, the role of oxidative stress in MDS has not been fully elucidated [55–57].

 Pellagatti et al. [50] investigated the expression profiles of a large group of ribosomal- and translation-related genes in the CD34^+^ cells isolated from bone marrow samples of 15 MDS patients with 5q-syndrome, 18 MDS patients with refractory anemia and a normal karyotype, and 17 healthy controls. Human genome U133 Plus 2.0 arrays (Affymetrix) covering over 47 000 transcripts representing 39 000 human genes were used in this study. The expression data for selected genes were validated using real-time quantitative PCR with predeveloped TaqMan assays (Applied Biosystems). The expression profiles of 579 probe sets for genes coding ribosomal proteins (229 for large 80S ribosomal subunit/RPL/and 176 for small 40S ribosomal subunit/RPS/) and for genes coding translation-related factors (149 for eukaryotic translation initiation factors/EIF/and 25 for eukaryotic translation elongation factors/EEF/) were analysed [50]. 55 genes were differentially expressed. 49 from these 55 genes (89%) showed lower expression in the 5q-syndrome patient group in comparison with MDS patients with refractory anemia and a normal karyotype and in comparison with healthy controls. These data were compared with data about the defective expression of genes for ribosomal proteins and translation-related factors in DBA published by Pellagatti et al. [50]. Three genes (*RPL28*,* RPS14*,* and EEF1D*) expression was found downregulated in both diseases (MDS 5q-syndrome and DBA) [50, 51]. In addition, the expression of two pro-3poptotic genes, *TNFRSF10B *(tumor necrosis factor receptor superfamily, member 10b gene) and *BAX *(BCL2-associated X protein gene), was upregulated in both diseases. Pellagatti et al. [50] suggested that the deregulation observed in ribosomal gene expression and translation-related gene expression in the CD34^+^ cells isolated from bone marrow samples of MDS patients with 5q-syndrome are secondary to RPS14 haploinsufficiency. These data and similar data of Sridhar et al. [52] support the hypothesis that the 5q-syndrome belongs to ribosomopathies, disorders of impaired ribosomal biogenesis (Figure 2).

## 3. The Role of miR-145 and miR-146a in the 5q-Syndrome

 In mammalian cells, miRNAs (microRNAs) are the most abundant family of small noncoding RNAs that regulate mRNA translation through the RNA interference pathway or target-specific mRNA for degradation [58]. In general, it appears that the major function of miRNAs is in development, differentiation, and homoeostasis, which is indicated by studies showing aberrant miRNA expression during the development of cancer.

 Starczynowski et al. [59] examined expression of microRNAs (miRNAs) encoded on chromosome 5q as a possible cause of haploinsufficiency. They showed that deletion of chromosome 5q correlates with loss of two miRNAs that are abundant in hematopoietic stem/progenitor cells (HSPCs), miR-145, and miR-146a. The miR-145 gene maps within the CDR of the 5q-syndrome [21], and the miR-146a gene lies adjacent to the distal boundary of the CDR and is also deleted in most patients with 5q-syndrome. Starczynowski et al. [59] identified Toll-interleukin-1 receptor domain-containing adaptor protein (TIRAP) and tumor necrosis factor receptor-associated factor-6 (TRAF6) as respective targets of these miRNAs. These miRNAs target corresponding genes through partial base pairing to the 3′-UTR of the target genes. These targets were aberrantly upregulated in 5q-syndrome [59]. Downregulation of the miRNAs (miR-145 and miR-146a) led to an upregulation of IL-6 that was dependent on TRAF6. Elevated IL-6 was found also in patients with 5q-syndrome [60]. TIRAP is known to lie upstream of TRAF6 in innate immune signaling. Knockdown of miR-145 and miR-146a together or enforced expression of TRAF6 in mouse HSPCs resulted in thrombocytosis, mild neutropenia, and megakaryocytic dysplasia. A subset of mice transplanted with TRAF6-expressing marrow progressed either to marrow failure or acute myeloid leukemia. Thus, inappropriate activation of innate immune signals in HSPCs phenocopies several clinical features of 5q-syndrome. Starczynowski et al. have recently showed that loss of two miRNAs, miR-145 and miR-146a, results in leukemia in a mouse model [61].

**Figure 3:**
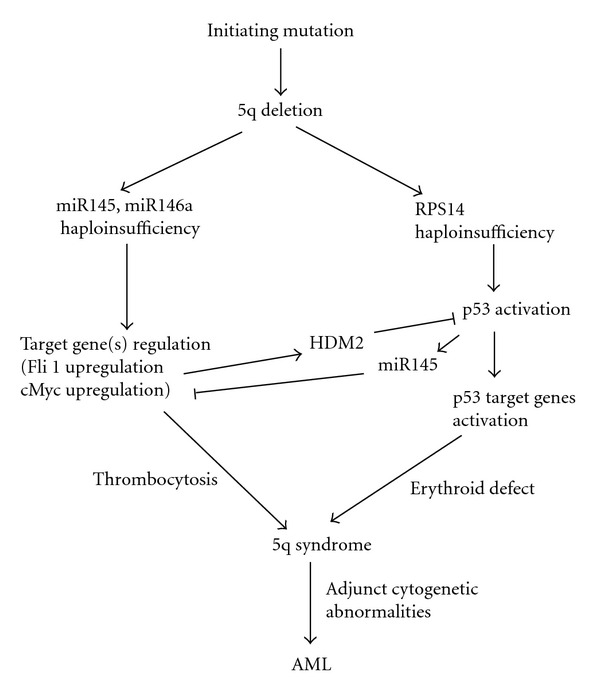
Schematic diagram of the development of the 5q-syndrome. The roles of the haploinsufficiency of RPS14, miR145, and miR146 and possible progression to AML by adjunct cytogenetic abnormalities are shown. Fli1 upregulation stimulates transcriptionally HDM2 [109] and inhibits partially p53 activation caused by ribosomal stress resulting from RPS14 haploinsufficiency.

 Patients with 5q-syndrome have decreased expression of miR-145 and increased expression of Fli-1 [62]. Overexpression of miR-145 or inhibition of Fli-1 in CD34^+^ cells decreases megakaryocyte production, while inhibition of miR-145 or overexpression of Fli-1 has the reciprocal effect. These findings have been validated *in vivo* using transgenic mice. Moreover, the combined loss of miR-145 and RPS14 cooperates to alter erythroid-megakaryocytic differentiation in a manner similar to the 5q-syndrome. Taken together, these findings demonstrated for the first time that coordinate deletion of a microRNA and a protein-coding gene (Figure 3) contributes to the phenotype of the 5q-syndrome [62].

 Other research groups found normal expression levels of miR-145 in most CD34^+^ cells isolated from bone marrow samples of MDS patients with 5q-syndrome [63, 64]. Remarkably, the guardians of the genome p53, p73, and p63 play a role in control of most of the known tumor suppressor miRNAs [65, 66]. Thus, it is possible that one allele of miR-145 is lost in 5q-syndrome but the second allele of miR-145 is overexpressed after p53 or related genes activation. A putative tumor suppressing miRNA, miR-145, has been shown to be decreased in various human cancers [67, 68], and it decreases the apoptosis and proliferation rate of colorectal cancer cells and B-cell lymphoma cell lines [69, 70].

 Zhang et al. [71] demonstrated that miR-145 targets a putative binding site in the 3′-UTR of the Friend leukemia virus integration 1 (Fli-1) gene and that miR-145 abundance is inversely related to Fli-1 expression in colon cancer tissues. Some other targets of miR-145 are important regulators of cell apoptosis and proliferation, such as c-Myc and IRS-1 [62, 72]. IRS-1, a docking protein for both the type 1 insulin-like growth factor receptor and the insulin receptor, delivers antiapoptotic and antidifferentiation signals. MiR-145 also downregulates the protooncogene c-Myc, whose aberrant expression is associated with aggressive and poorly differentiated tumors. Zhang et al. [73] demonstrated that DNA fragmentation factor 45 (DFF45) expression is controlled at the translational level by miR-145, using bioinformatic and proteomic techniques. DFF45 is caspase-3 or caspase-7 substrate that must be cleaved before apoptotic DNA fragmentation can proceed [74, 75].

 Changes in the expression of miR-146a have been implicated in both the development of multiple cancers and in the negative regulation of inflammation induced via the innate immune response. Furthermore, miR-146a expression is driven by the transcription factor NF-*κ*B (nuclear factor kappaB), which has been implicated as an important causal link between inflammation and carcinogenesis. Williams et al. [76] and Li et al. [77] reviewed the evidence for a role of miR-146a in innate immunity and cancer and assessed whether changes in miR-146a might link these two biological responses.

 The role of miR-146a in hematopoiesis was investigated by using retroviral infection and overexpression of miR-146a in mouse hematopoietic stem/progenitor cells, followed by bone marrow transplantations [78]. miR-146a is mainly expressed in primitive hematopoietic stem cells and T lymphocytes. Overexpression of miR-146a in hematopoietic stem cells, followed by bone marrow transplantation, resulted in a transient myeloid expansion, decreased erythropoiesis, and impaired lymphopoiesis in select anatomical locations. Enforced expression of miR-146a also impaired bone marrow reconstitution in recipient mice and reduced survival of hematopoietic stem cells. These results indicate that miR-146a, a lipopolysaccharide- (LPS-) induced miRNA, regulates multiple aspects of hematopoietic differentiation and survival. Furthermore, the consequences of miR-146a expression in hematopoietic cells mimic some of the reported effects with acute LPS exposure.

 Third gene for miRNA in the CDR is *miR-143* [62]. Cordes et al. [79] and Boettger et al. [80] proposed that miR-145 and miR-143 regulate smooth muscle cells (SMC) fate and plasticity and play the important role in cardiovascular development. MDS patients with 5q deletion were characterized by decreased levels of miR-143 and miR-378 mapped within the commonly deleted region at 5q32 [81].

## 4. The Importance of p53 in the Molecular Mechanism of 5q-Syndrome

 Dysregulation of ribosome biogenesis through insufficiency of ribosomal subunits has been demonstrated to activate a p53 checkpoint and regulate developmental pathways [25, 82–86]. The p53 pathway provides a surveillance mechanism for translation in protein synthesis and for genome integrity. The transcription factor p53 is encoded by *TP53* tumor suppressor gene. Target genes of p53 regulate critical processes, such as apoptosis, cell cycle arrest, DNA repair, senescence, and cellular metabolism.

### 4.1. Activation of p53 by Ribosome Dysfunction in Model Systems

 Activation of p53 stimulates proteasome-dependent truncation of eIF-4E-binding protein 1 (4E-BP1) [87]. The translational inhibitor protein 4E-BP1 regulates the availability of polypeptide chain initiation factor eIF4E for protein synthesis. Initiation factor eIF4E binds the 5′ cap structure present on all cellular mRNAs. Its ability to associate with initiation factors eIF4G and eIF4A, forming the eIF4F complex, brings the mRNA to the 43S complex during the initiation of translation. Binding of eIF4E to eIF4G is inhibited in a competitive manner by 4E-BP1. Phosphorylation of 4E-BP1 decreases the affinity of this protein for eIF4E, thus favouring the binding of eIF4G and enhancing translation. The truncated protein 4E-BP1 is almost completely unphosphorylated and exerts a long-term inhibitory effect on the availability of eIF4E, thus contributing to the inhibition of protein synthesis and the growth-inhibitory and proapoptotic effects of p53.

 The murine double minute 2 protein (MDM2) and human analogue (HDM2) function as a link between ribosome biogenesis and the p53 pathway. MDM2 or HDM2 proteins function in two major ways: (1) as an E3 ubiquitin ligase that recognizes the N-terminal transactivation domain (TAD) of the p53 tumor suppressor and (2) as an inhibitor of p53 transcriptional activation [88–90]. Having a short half-life, p53 is normally maintained at low levels in unstressed mammalian cells by continuous ubiquitination and subsequent degradation by the 26S proteasome. This is primarily due to the interaction of p53 with the RING-finger ubiquitin E3 ligases, MDM2 or HDM2. MDM2 interacts with a variety of ribosomal proteins, including RPL5, RPL11, RPL23, RPL26, and RPS7 [91–99]. These interactions, which typically involve the acidic domain and sometimes the adjacent zinc finger of MDM2, interfere with the inhibitory functions of this region of MDM2 and contribute to p53 activation (Figure 2). As first exemplified for RPL11 [92], these interactions increase when ribosome biogenesis is disrupted, in a situation termed “ribosomal biogenesis stress” or “nucleolar stress” [100, 101]. Mechanistically, ribosomal stress causes translocation of free ribosomal proteins from the nucleolus to the nucleoplasm [94, 102], where they bind MDM2 [94]. The increased binding of ribosomal proteins to MDM2 augments cellular p53 activity, leading to growth arrest and coupling deficient protein synthesis with cessation of cell proliferation.

 However, it is not clear whether ribosomal protein regulation of MDM2 is specific to some, but not all ribosomal proteins. Sun et al. [103] showed that RPL29 and RPL30, two ribosomal proteins from the 60S ribosomal subunit, do not bind to MDM2 and do not inhibit MDM2-mediated p53 suppression, indicating that the ribosomal protein regulation of the MDM2-p53 feedback loop is specific.

### 4.2. Activation of p53 and Upregulation of the p53 Pathway in 5q-Syndrome

 Results of several laboratories described in Section 4.1 showed that upregulation of the p53 pathway is a common response to haploinsufficiency of ribosomal proteins. Pellagatti et al. [104] carried out the analysis of the p53 pathway in the 5q-syndrome. They used Affymetrix arrays [50]. Ten genes in the p53 pathway (*FAS*/tumor necrosis factor receptor superfamily, member 6 gene/*, CD82*/cluster of differentiation 82 gene/,* WIG1*/wild type p53-induced gene 1 gene/, *CASP3*/cysteine-aspartic acid protease, caspase 3 gene/, SESN3/sestrin 3 gene/,* TNFRSF10B*,* MDM4*/murine double minute 4 gene/,* BAX*,* DDB2*/damage-specific DNA binding protein 2 gene/, and *BID2/*BH3-interacting domain death agonist 2 gene/) were significantly deregulated. All these genes with exception of *MDM4 *(negative regulator of p53, structurally related to MDM2 [105]) were expressed at higher levels in CD34^+^ cells from 5q-syndrome patients in comparison with healthy controls. *MDM4 *is expressed at lower level in 5q-syndrome patients. Further upregulated genes in 5q-syndrome (*RPS27L*/ribosomal protein S27-like gene/*, PHLDA3*/pleckstrin homology-like domain, family A, member 3 gene/,* BCL11B*/B-cell lymphoma/leukemia 11B gene/, and* FDXR*/ferredoxin reductase gene/) are p53 targets [21, 50]. Strong p53 expression was confirmed not only on the mRNA level but also on the protein level by an immunohistochemical analysis. Activation of p53 as a result of ribosomal stress seems to play an important role in the 5q-syndrome. Upregulation of the p53 pathway offers a new potential therapeutic target in the human 5q-syndrome. This therapy is possible only if the critical tumor suppressor function of p53 will be preserved.

### 4.3. The Relation between p53 and miR145

 The tumor suppressor p53 is a master gene regulator capable of regulating numerous genes. Since p53 plays a critical role in suppression of cell growth and proliferation, it is considered as a complementary opposite factor to the oncoprotein c-Myc. The oncoprotein c-Myc can counteract the p53's action to a great degree. The cell has developed sophisticated mechanisms during evolution to balance the effect of both these factors. It has been known for a long time that there is a negative correlation between p53 and c-Myc. Although previous report [106] suggested that this might involve transcriptional repression the precise mechanism of p53-mediated repression of c-Myc is not fully understood. Sachdeva et al. [107] have recently demonstrated that p53 induces *miR-145 *and subsequently represses c-Myc. Thus, identification of *miR-145*, that was described in Section 3 as a new player in this p53 regulatory network (Figure 3), provides at least one aspect of how the cell balances the effects of p53 and c-Myc [108].

### 4.4. TP53 Mutation in 5q-Syndrome

 TP53 mutations were often detected in high-risk MDS and AML with del(5q) [110, 111]. Jädersten et al. [112] described a patient with 5q-syndrome with del(17p) and *TP53* mutation at disease progression. They demonstrated the *TP53* mutant clone already at the initial diagnosis. Consequently, they analysed 55 patients with low- and int-1 risk MDS and del(5q) and identified 10 patients (18%) with *TP53* mutations [113]. Moreover, they found a correlation between TP53 mutation and strong nuclear p53 protein expression in bone marrow progenitors [113]. Patients with mutations had significantly worse outcome. Clonal heterogeneity in low-risk 5q-syndrome patients may be of importance for the prognosis and therapy [113].

## 5. Role of Further Genes in 5q-Syndrome

### 5.1. The Role of Other Genes Positioned on 5q Chromosome

 The murine model for the 5q-syndrome with haploinsufficiency of the *Cd74*-*Nid67* region described in the Section 2.2 showed that other gene in this region may play an additional role. *Dctn4 *gene encodes Dynactin 4, a subunit of the dynactin complex. The dynactin complex binds cargo, such as vesicles and organelles, to cytoplasmic dynein for retrograde microtubule-mediated trafficking [114]. Retroviral expression in megakaryocytes of dynamitin (p50), which disrupts dynactin-dynein function, inhibits proplatelet elongation [115]. Patel et al. [115] concluded that while continuous polymerization of microtubules is necessary to support the enlarging proplatelet mass, the sliding of overlapping microtubules is a vital component of proplatelet elongation. Thus, the inhibition of proplatelet elongation might reduce platelet formation and lead to thrombocytopenia [116]. The further gene *Rbm22* was described in Section 2. RBM22 belongs to the SLT11 family; it has been reported to be involved in prespliceosome assembly and to interact with the Ca^2+^-signaling protein ALG-2 [117]. RBM22 is underexpressed in 5q-syndrome and deregulates probably apoptosis [29].

 In addition to the minimal CDR on chromosome 5q33.1 in 5q-syndrome, a second, proximal CDR on chromosome 5q31.2 contains genes *EGR1*,* CTNNA1* (gene-encoding alpha-catenin), and *HSPA9 *(gene-encoding mortalin, GRP75, PBP74, and MTHSP75). Loss of this CDR contributes to a more aggressive MDS or AML phenotype [16, 17].

 The gene for nucleophosmin (*NPM1*) localized on chromosome 5q35.1 (Figure 1) is deleted in many cases of 5q- MDS. Targeted knockout of one allele of *NPM1* leads to genetic instability [118–120]. *NPM1* heterozygous mice develop both myeloid and lymphoid malignancies. The bone marrow of these mice is hypercellular with evidence of dysplasia of the erythroid and megakaryocytic lineages. Immature erythroid cells accumulate in this bone marrow. The *NPM1 *haploinsufficiency in patients with large 5q deletions contributes to the phenotype of MDS. Moreover, *NPM1* is the most commonly mutated gene in patients with normal karyotype AML [121–123].

### 5.2. The Role of Genes Not Positioned on Chromosome 5

 We studied the role of the levels of mRNA for transcriptions factors Fli-1 (Friend leukemia virus integration 1) and EKLF (erythroid Krűppel-like factor, also named KLF1) in mononuclear cells isolated from bone marrow and peripheral blood of MDS patients with 5q-syndrome in comparison with patients with low risk MDS without 5q chromosome abnormality and with healthy controls [124, 125]. We found increased Fli1 mRNA levels and decreased EKLF mRNA levels in 5q-syndrome. The decreased expression of miR-145 might be responsible for increased Fli-1 levels in 5q-syndrome (see Section 3). We suggest that increased Fli-1 expression in mononuclear cells of MDS patients with 5q-syndrome is further stimulated by the increased Fli-1 level and by the decreased EKLF level.

 Fli-1 is a member of the Ets family of transcription factors. Fli-1 was originally identified as a protooncogene in Friend virus-induced erythroleukemia in mice [126]. The founding member of this family, the oncogene v-ets, was discovered in the genome of avian leukosis virus E26. Ets transcription factors bind to DNA elements containing the consensus sequence GGA(A/T). Fli-1 is preferentially expressed in hematopoietic cells and in vascular endothelial cells. Pulse-field gel analysis has localized the Fli-1gene within 240 kb of the Ets-1 locus on mouse chromosome 9 and on human chromosome 11q23, suggesting that these two ets genes arose by gene duplication from a common ancestral gene [127, 128]. Human Fli-1 contains nine exons which extend over approximately 120 kb [129].

 Fli-1 plays an important role during the normal development of megakaryocytes. *Fli-1* knockout mice produce small, undifferentiated megakaryocytic progenitors with abnormal features. Fli-1 represses the transcription of EKLF gene [130] and activates the promoters of several megakaryocyte-specific genes (glycoprotein IX and thrombopoietin receptor genes promoters) [131, 132]. Fli-1 gene promoter is upregulated by Fli-1 and other Ets factors (Ets1, Ets2, and Elf1) but is inhibited by Tel [133]. Transcription factor PU.1 (also known as Spi-1) is also a positive regulator of the *Fli1* gene [134]. On the other hand, PU.1 inhibits transcription factor GATA-1 function and erythroid differentiation by blocking GATA-1 DNA binding [135, 136]. Both, PU.1 and Fli-1 directly activate common target genes involved in ribosome biogenesis [137]. One of the consequences of miR145 and miR146a depletion or TIRAP and TRAF6 activation in the innate immune system pathway is overexpression of IL-6 which activates Fli-1 gene expression [59, 78, 138–140].

 EKLF (erythroid Krűppel-like factor, also named KLF1) is a zinc finger transcription factor that plays a prominent role during erythroid development [141–143]. Human gene *EKLF *is positioned on chromosome 19p13.12-13 [144]. Commitment towards megakaryocyte versus erythroid blood cell lineages occurs in the megakaryocyte-erythroid progenitor (MEP). EKLF restricts megakaryocytic differentiation to the benefit of erythrocytic differentiation, and this effect is mediated by the inhibition of Fli-1 recruitment to megakaryocytic and *Fli-1* genes promoters [130, 145–147]. A marked increase in the number of circulating platelets in *EKLF* null mouse was found [148].

## 6. Conclusions and Perspectives

 Candidate genes were studied using gene deletions or similar approaches to determine whether they have a role in 5q-syndrome, a subtype of MDS. A major advance occurred in 2008 when RNA interference-based approach for all 40 genes located within the 5q- CDR was used to discover the 5q- disease gene. A defect in the function of a ribosomal protein RPS14 that is important for the proper processing of the 40S ribosomal subunit has been implicated in the pathophysiology of the 5q-syndrome. The *RPS14 *gene is located in the deleted region, and it seems that this loss affects erythroid differentiation. Haploinsufficiency of *RPS14 *gene in 5q-syndrome is associated with deregulation of ribosomal- and translation-related genes. Dutt et al. [149] found that p53 accumulates selectively in the erythroid lineage in primary human hematopoietic progenitor cells following expression of shRNAs targeting RPS14 or RPS19. Thus, 5q-syndrome is similarly as Diamond Blackfan anemia, where *RPS 19* is the most commonly mutated gene, a ribosomopathy [25, 34, 36, 150, 151].

 Induction of p53 in both diseases, but not in control samples, led to lineage-specific accumulation of p21^WAF1, CIP1^, also known as cyclin-dependent kinase inhibitor 1 or CDK-interacting protein 1 (a proliferation arrest determinating protein that in humans is encoded by the *CDKN1A* gene located on chromosome 6p21.2). The induction of p21^WAF1, CIP1^ caused cell cycle arrest in erythroid progenitor cells. Pharmacological inhibition of p53 rescued the erythroid defect, while a compound that activates p53 through inhibition of HDM2, nutlin-3, selectively impaired erythropoiesis [149]. The increased apoptosis was observed in the 5q- mouse bone marrow [35]. In response to diverse stresses, the tumor suppressor p53 differently regulates its target genes, inducing cell cycle arrest, apoptosis, or senescence. The p53 is critical regulator of hematopoietic stem cell (HSC) behavior and plays an important role in regulating HSC quiescence, self-renewal, apoptosis, and aging. The p53 promotes HSC quiescence [152, 153]. The mutant p53 protein can provide escape from apoptosis and facilitates also the entry of HSC to the cell cycle. Mutations in *TP53* are relatively uncommon in cases of primary (*de novo*) MDS but have high incidence in patients with therapy-related MDS [154–156]. Garderet et al. [157] found defective erythroid proliferation but not differentiation capacity related to *RPS14 *gene haploinsufficiency. They used *in vitro *model of erythropoiesis [158, 159] in which mature red blood cells are generated from human progenitor cells to analyze cell proliferation and differentiation in a homogeneous erythroid population with *RPS14 *gene haploinsufficiency in culture. The enucleation capacity of 5q- clones remained unchanged. Therefore, the decreased erythroid maturation and subsequent anemia seen in 5q-syndrome are unlikely caused by a specific blockade of late differentiation and are probably the consequences of the proliferative defect of both pathological and residual nondeleted clones in the patient′s bone marrow [160].

Treatment of zebrafish models of ribosomopathies with L-leucine results in an improvement of anemia and developmental defects in both diseases (Diamond Blackfan anemia and 5q-syndrome) [161]. Cmejlova et al. [162] demonstrated that the efficiency of mRNA translation is significantly depressed in cells derived from Diamond Blackfan anemia patients, consistent with a pathogenic ribosomal defect. L-leucine is an essential branched chain amino acid that is known to modulate protein synthesis by enhancing translation [162–166]. L-leucine has been used to treat Diamond Blackfan anemia patients [162, 167]. Treatment of *RPS19*, and *RPS14-*deficient zebrafish embryos and human hematopoietic progenitor CD34^+^ cells with L-leucine resulted in partial reversal of both the anemia and the developmental defects and provided evidence for a common signaling pathway involving the mTOR (mammalian target of rapamycin) integrating growth-promoting and stress signals [161].

 From a translational point of view, the identification of the genes and miRNAs involved in the pathogenesis of the 5q-syndrome may support the development of novel targeted therapeutic strategies. The immunomodulatory drug lenalidomide (CC-5013; REVLIMID, Celgene Corporation), a structural analogue of thalidomide, is indicated for the treatment of the 5q-syndrome MDS patients, rendering 67% of patients transfusion independent and inducing cytogenetic responses in over 40% of them [168–173]. In contrast, in a large multicenter trial involving transfusion: dependent MDS patients without del(5q) only 26% achieved transfusion independence with infrequent cytogenetic improvement [174]. The selective and specific activity of lenalidomide in MDS remains undefined. Gene expression profiling and disease- and therapy-associated proteome changes in the sera of MDS patients were used for monitoring and predicting the response to therapy [175, 176]. Wei et al. [177] provided evidence that lenalidomide is selectively cytotoxic to del(5q) cells as a result of inhibition of the haplodeficient dual specificity phosphatases, Cdc25C and PP2A. Phosphorylation of MDM2 and Fli-1 is important for the level and the activity of both these proteins involved in the process of erythroid failure in 5q-syndrome, and the level of PP2A regulates accumulation and degradation of p53 and Fli-1 [178, 179]. The mechanism of action of lenalidomide is complex and includes inhibition of a wide array of proinflammatory cytokines, such as interleukin-6 (IL-6), and upregulation of T-helper-secreted cytokines, such as IL-2 and IFN-gamma [180]. It has been also described that IL-2 subsequently activates natural killer (NK) cells. In view of the recently revealed role of IL-6 in the pathogenesis of the 5q-syndrome [59, 138], it is tempting to speculate that the responses induced by lenalidomide therapy in patients with this disorder are due to downregulation of IL-6. Therefore, IL-6 expression and/or upstream regulators, such as TRAF6, may represent targets, which suggests that agents capable of inhibiting the TRAF6-IL-6 axis may be clinically useful for the management of 5q-syndrome [59, 138].

 Lenalidomide inhibits the malignant clone and upregulates the *SPARC *and *RPS14 *genes expression [181, 182]. Both these genes are localized in CDR on chromosome 5. Induction of miR-143 and miR-145 in CD34^+^ cells of MDS patients with the del(5q) abnormality after exposure to lenalidomide correlates with clinical response [179]. The beneficial effect of lenalidomide in patients with 5q-syndrome is associated with significant increases in the proportion of bone marrow erythroid precursor cells and in the frequency of clonogenic progenitor cells, a substantial improvement in the hematopoiesis-supporting potential of bone marrow stroma and significant alterations in the adhesion profile of bone marrow CD34^+^ cells [183]. Although lenalidomide efficiently reduced a larger fraction of the del(5q) stem cells, some rare and phenotypically distinct quiescent del(5q) stem cells remained resistant to lenalidomide [112, 184]. Over time, lenalidomide resistance developed in most of the patients with recurrence or expansion of the del(5q) clone and clinical and cytogenetic progression. The resistant clone, insensitive to lenalidomide, overexpresses p53 and sequencing confirmed *TP53 *mutation [185]. The presence of easily detectable subclones with inactivated p53, and thereby a more malignant potential, is important and has prognostic value.
